# Methylammonium Polyiodides in Perovskite Photovoltaics: From Fundamentals to Applications

**DOI:** 10.3389/fchem.2020.00418

**Published:** 2020-05-13

**Authors:** Andrey A. Petrov, Alexey B. Tarasov

**Affiliations:** ^1^Laboratory of New Materials for Solar Energetics, Department of Materials Science, Lomonosov Moscow State University, Moscow, Russia; ^2^Department of Chemistry, Lomonosov Moscow State University, Moscow, Russia

**Keywords:** methylammonium polyiodides, reactive polyiodide melts, RPM, hybrid perovskites, lead halide perovskites, perovskite solar cells

## Abstract

Discovered in 2017, methylammonium polyiodides were proposed as a facile precursor for synthesis of hybrid perovskites by means of their interaction with metallic lead, which initiated further active exploration of their potential applications. Investigation of their unusual properties such as liquid state, unprecedented phase diversity and high reactivity revealed that methylammonium polyiodides are the first representatives of a new class of compounds—reactive polyhalide melts (RPM). In this review, we summarize the reported data on the unique properties of these compounds, discuss their potential for fabrication of hybrid perovskite films and describe the role of polyhalides in degradation of perovskite solar cells.

## Introduction

Hybrid organic-inorganic perovskites with a general formula APbX_3_ [A = CH_3_${\text{NH}}_{3}^{+}$ (MA^+^), CH(NH_2_)${}_{2}^{+}$ (FA^+^); X = I^−^, Br^−^] represent a new perspective class of materials used for optoelectronic devices (LEDs, lasers, sensors, etc.) and solar cells of new generation, so called perovskite solar cells. For the last decade, unprecedented interest to these materials has arouse because of the unique combination of their properties such as strong light absorption (Lin et al., 2015), large electron and hole diffusion lengths (Dong et al., 2015), wide tunability of their properties through the selection of the composition and facile fabrication using solution-processing methods (Gao et al., 2014; Zhao and Zhu, 2016). The efforts applied to the development of perovskite solar cells lead to an outstanding growth of their efficiency from ~4% in 2009 to 25.2% in 2019 (NREL, 2020) thus overcoming record efficiencies for silicon solar cells.

Almost all proposed methods for fabrication of perovskite films rely on one of two processes: crystallization of hybrid perovskites from polar aprotic solvents (Saliba et al., 2016; Kim et al., 2019) or addition reaction between lead salt and organic salt (e.g., PbI_2_ and MAI) (Yang et al., 2017). While deposition from solutions remains the most common method for obtaining high-quality perovskite films due to its simplicity, the crystallization of perovskites goes through formation of solvate intermediate phases such as (MA)_2_(S)_2_Pb_3_I_8_ (S = DMSO, DMF, GBL) which tend to grow in a form of needle-like crystals resulting in inhomogeneous morphology and pinholes of perovskite films (Cao et al., 2016; Petrov et al., 2017b,c; Fateev et al., 2018). The process is further exacerbated with the shrinkage of film volume upon decomposition of the intermediate phases into perovskite leading to the formation of cracks. The approaches based on reaction between lead and organic salts can be implemented in two ways: either by (co-)evaporation in vacuum or using successive solution deposition. The former needs a complex vacuum equipment whereas the latter suffers from the incomplete reaction (Hsieh et al., 2017). The abovementioned issues provoked search and development of alternative synthesis methods.

The recent discovery of a new group of low-melting methylammonium and formamidinium polyiodides (Petrov et al., 2017a) opened a new branch of strategies for hybrid perovskite synthesis through a redox reaction with metallic lead. Because of their liquid state and high reactivity toward metallic lead, the name of such polyiodides were coined as reactive polyiodide melts (RPM). In present review, we give a generalized vision on methylammonium polyiodides and related compounds known so far in terms of their fundamental features and technological applications.

## Liquid State

**Figure 1:**
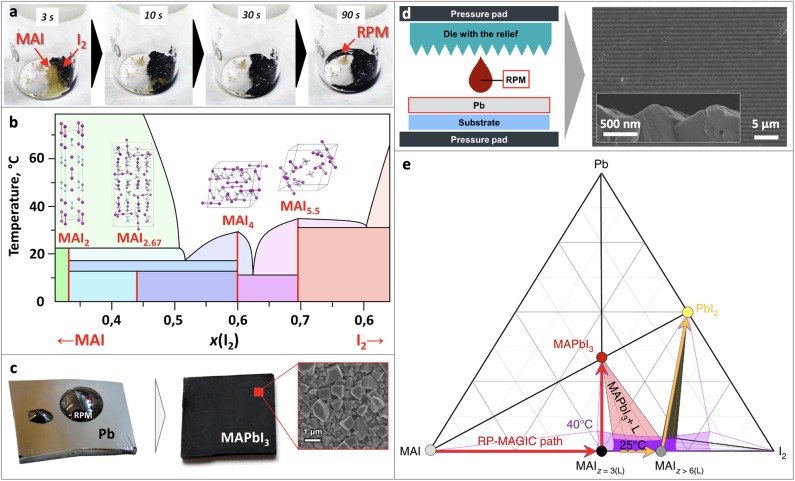
**(a)** Formation of RPM from MAI and I_2_, **(b)** Phase diagram of MAI-I_2_, **(c)** perovskite film obtained by spin-coating of RPM onto Pb film, **(d)** scheme of a method for fabrication textured perovskite films, **(e)** Ternary Pb-MAI-I_2_ diagram. Adapted from Petrov et al. (2017a) with permission from the Royal Society of Chemistry. Adapted with permission from Petrov et al. (2019). Copyright (2019) American Chemical Society. Adapted from Grishko et al. (2019) with permission from the Royal Society of Chemistry. Material from: “Ivan Turkevych et al. Strategic advantages of reactive polyiodide melts for scalable perovskite photovoltaics. Nature Nanotechnology, published 2018, Springer Nature”.

RPM can be easily obtained by simple mixing of crystalline methylammonium iodide (MAI)/formamidinium iodide (FAI) and iodine (I_2_) (Petrov et al., 2017a). If the two solid compounds are brought into contact, a liquid RPM immediately forms even at room temperature (Figure 1a). Similarly, a crystal of organic salts transforms into RPM droplet in the atmosphere containing I_2_ vapors (Turkevych et al., 2019).

According to Raman spectroscopy, methylammonium polyiodides have three characteristic vibrations near 110 cm^−1^, 145 cm^−1^ and 170 cm^−1^ that correspond to an ${\text{I}}_{3}^{-}$ symmetrical stretch vibrations, ${\text{I}}_{3}^{-}$ asymmetrical stretch vibrations and vibrations of solvating I_2_ molecular units, respectively (Petrov et al., 2017a; Turkevych et al., 2019; Udalova et al., 2020).

Liquid state of the RPM is a distinctive feature, making it a special precursor for hybrid perovskites because it does not require any organic solvents and therefore exempts the fabrication process from concomitant problems. In fact, a liquid state of RPM at room temperature is not unique itself if considering the whole class of plolyiodides. However, it was previously thought that only polyiodides with large organic cations such as aromatic (e.g., imidazolium) (Thorsmølle et al., 2012; Fei et al., 2015), trialkylsulfonium [e.g., (Et)_3_S^+^] (Bengtsson et al., 1991), or tetraalkylammonium [e.g., (Oc)_4_N^+^] (Stegemann et al., 1992; Wang et al., 2014; Yushina et al., 2014) are able to generate liquid phases of such polyiodides at room temperature (Bengtsson et al., 1991; Stegemann et al., 1992; Thorsmølle et al., 2012). Conversely, CsI_3_ has a melting point over 130°C (Topol, 1968).

The melting points of methylammonium and formamidinium polyiodides were found to be unexpectedly low. Detailed physicochemical analysis of the RPM revealed a complex phase diagram of the MAI-I_2_ system with 3 eutectics and 4 polyiodide phases MAI_2_, MAI_2.67_, MAI_4_, MAI_5.5_ (Figure 1b). While MAI_2_ and MAI_2.67_ phases were found to undergo peritectic and peritectoid decompositions at 23°C and 14°C, the melting temperatures of MAI_4_ and MAI_5.5_ phases were determined at 35°C (Petrov et al., 2019).

To estimate thermodynamic stability of the crystalline polyiodides, DFT calculations were performed. The selected approach to calculate formation enthalpy and entropy values of the compounds from isolated particles (I^−^, MA^+^, I_2_) demonstrated excellent agreement with experimental data for MAI and I_2_ and was therefore used to calculate thermodynamic state-functions of the polyiodides. The performed DFT calculations revealed that MAI_2_ forms due to major enthalpy contribution whereas in the case of higher methylammonium polyiodides entropy plays a crucial role in their formation (Petrov et al., 2019).

The low melting points of methylammonium polyiodides were explained by high energy of cation solvation and increase in conformational entropy upon melting. The former arises from high dipole moment of methylammonium cation along with its ability to form numerous H-bonds therefore ensuring strong interaction with anions in a liquid state. The latter follows from the low energy barriers to rotation of the cations and their amphiphility leading to the realization of all possible cation-anion conformations in the melt (Petrov et al., 2019).

According to the phase diagram, there are two compositional regions with a liquid homogeneous state of MAI_x_ at room temperature (3.0 < x <3.3; 4.3 < x <4.4), whereas at 40°C a liquid state of MAI_x_ is observed in the wide range of compositions (2.9 < x < 9.0), which opens up great opportunities for technological application of RPM.

## High Reactivity

The RPM demonstrate high reactivity toward various metals. In particular, the reaction of RPM with metallic lead manifests an amazingly simple redox process of perovskite synthesis:

MAI_3(liquid)_ + Pb _(solid)_ -> MAPbI_3__(solid)_

The process can be implemented by spin-coating or spraying of RPM onto Pb films. If such treatment is followed by rinsing of the specimen with isopropanol to eliminate the unreacted RPM, large cuboid morphology of the film can be observed (Figure 1c). In case of the incomplete conversion there is PbI_2_ layer between the layer of unreacted Pb and grown perovskite layer according to XRD and SEM data. On the other hand, long contact of RPM with perovskite film leads to its recrystallization and dissolution. For instance, it was shown, that 50 nm thickness film of lead treated with RPM completely disappear within 15 s indicating that some amount of lead can be dissolved in RPM. Later experiment showed that the solubility of Pb is rather small and does not exceed 1 mol.% at room temperature and slightly increases at elevated temperatures.

The conversion of Pb into MAPbI_3_ is accompanied by volume increase in 8.4 times thus ensuring the formation of pinhole free perovskite films. This feature was successfully utilized for perovskite growth in confined space. Thin patterned perovskite films were obtained by pressing the die with a given relief containing RPM deposited onto it against the metallic lead film (Figure 1d). Because the sum of molar volumes of Pb and RPM is almost equal to that for MAPbI_3_, the proposed method allows to dose the exact amount of RPM and fabricate perovskite films with any particular relief (Grishko et al., 2019), which can improve the efficiency of the devices due to optimization of light-scattering (Wang et al., 2012). The other way to dose RPM can be the use of starch to eliminate its excess through formation of RPM-starch complex (Shlenskaya et al., 2018b).

Other metals such as Cu and Au are also subject to the reaction with RPM resulting in formation of MACu_2_I_3_ and (MA)_2_Au_2_I_6_, respectively (Petrov et al., 2018; Shlenskaya et al., 2018a). Likewise, RPM easily oxidize spiro-MeOTAD which is the most popular material for hole-transporting layer in perovskite solar cells (Shlenskaya et al., 2018a). The formation of these products is particularly important when one considers stability of perovskite solar cells and formation of RPM under operational conditions in the perovskite-based devices, which is discussed in the last section.

## Phase Relations in the Pb-MAI-I_2_ Ternary System

Phase diagram of the Pb-MAI-I_2_ system (Figure 1e) shows the technologically relevant area, where RPM coexist with MAPbI_3_ (light-red triangle), which enlarges with the temperature increase. On the right side of it there is an unfavorable area in which iodine-rich RPM coexist with PbI_2_. A reaction between Pb and RPM corresponds to a linear pathway on the phase diagram from the point corresponding to a particular composition of RPM toward Pb apex. In order to obtain pure perovskite phase, one should develop a synthetic process which does not go beyond the light-red area. Therefore, RPM should have the composition MAI_X_, where x < 5.5 to avoid PbI_2_ formation and x > 3 (for the case of T = 40°C) to be liquid for ensuring effective lead conversion (Petrov et al., 2017a; Turkevych et al., 2019).

## Perovskite Solar Cells

Although it is tricky to dose RPM precisely onto metallic lead due to its extremely high reactivity, two approaches were proposed to solve this task. The first is based on addition of a reagent which initiates RPM formation and further reaction, whereas the second is based on withdrawal of a compound which inhibits the reaction.

**Figure 2:**
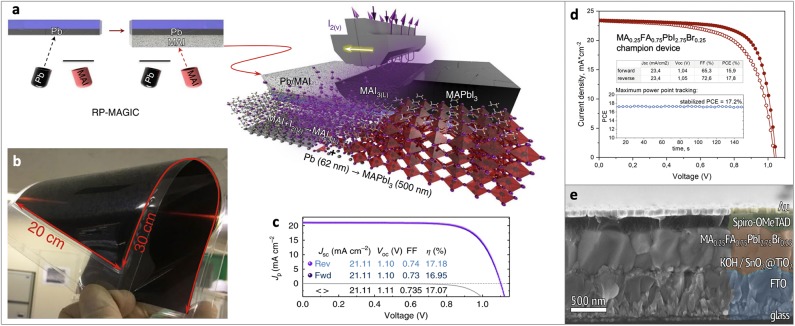
**(a)** Scheme of the RP-MAGIC approach, **(b)** MAPbI_3_ film on a 20 × 30 cm^2^ flexible PET/ITO substrate obtained by RP-MAGIC approach, **(c)** PCE of the device obtained by RP-MAGIC approach, **(d,e)** PCE and cross-section of the device obtained by spin-coating of RPM solution onto Pb film. Material from: “Ivan Turkevych et al. Strategic advantages of reactive polyiodide melts for scalable perovskite photovoltaics. Nature Nanotechnology, published 2018, Springer Nature”. Adapted with permission from Belich et al. (2020). Copyright (2020) American Chemical Society.

The first approach is named RP-MAGIC (Reactive Polyiodide Melt-Assisted Growth through *in-situ* Conversion), in which a stoichiometric Pb/MAI bilayer on a substrate obtained by subsequent thermal evaporation of Pb and MAI is treated with iodine vapors at 40°C (Figure 2a). Upon the reaction of MAI_(solid)_ and I_2(gas)_ the RPM is formed and instantly reacts with a Pb underlayer:

Pb _(solid)_ + MAI _(solid)_ + I_2(gas)_ -> MAPbI_3__(solid)_

Authors demonstrated highly uniform perovskite films on relatively large area such as 100 cm^2^ on glass/FTO and 600 cm^2^ on flexible PET/ITO substrates therefore showing high scalability potential of the method (Figure 2b). The solar cells fabricated using RP-MAGIC approach demonstrated power conversion efficiencies (PCE) up to 17.2% (reverse scan) in the standard FTO/m-TiO_2_/Perovskite/Spiro-OMeTAD/Au architecture (Figure 2c) (Turkevych et al., 2019).

Another approach is a spin-coating onto Pb film a certain amount of RPM dissolved in isopropanol. The reactivity of RPM in such solution was shown to be inhibited by the presence of solvent molecules. Upon evaporation of isopropanol, pure RPM is formed starting to react with the lead film. Using this method high-quality perovskite films were obtained and perovskite solar cells with PCE = 17.8% (reverse scan) were demonstrated (Figures 2d,e) (Belich et al., 2020).

Although the obtained efficiency of solar cells obtained using RPM is less than that achieved by conventional solution based spin-coating approach with record values up to 21% for MAPbI_3_ (Chiang and Wu, 2018; Liu et al., 2019b), the first proof-of-concept results obtained without advanced optimization of charge transporting layers are fair enough and comparable with other alternative approaches such as crystallization from methylamine/acetonitrile mixture (19.0%) (Noel et al., 2017) methylamine based technology (19.3%) (Chen et al., 2017).

To achieve the efficiencies over 22%, mixed-cation and mixed-halide perovskites should be used (Yang et al., 2017; Jiang et al., 2019; Jung et al., 2019; Kim et al., 2019; Liu et al., 2019a). Such technologically attractive perovskites with mixed compositions were also successfully obtained using the proposed methods as the corresponding polyiodides have melting points below 65°C (Petrov et al., 2019; Turkevych et al., 2019). In particular, Cs_0.05_MA_0.2_FA_0.75_PbI_3_ perovskite films and corresponding devices were obtained by RP-MAGIC approach [T_m_(Cs_0.05_MA_0.2_FA_0.75_I_3_) = 38°C] (Turkevych et al., 2019). Mixed compositions [MA_0.5_FA_0.5_PbI_3_, MA_0.25_FA_0.75_PbI_3_, MA_0.25_FA_0.75_PbI_2.75_Br_0.25_, (FAPbI_3_)_0.83_(MAPbBr_3_)_0.17_] were obtained by spin-coating of the RPM solutions onto Pb films (T_m_ of all corresponding polyhalides < 5°C) (Petrov et al., 2019; Belich et al., 2020). Further optimization of polyhalide compositions as well as their application techniques is therefore a vital scientific task which can further improve the efficiency of the devices obtained using RPM.

## Formation of RPM Under Illumination

It is known that intensive irradiation of hybrid perovskites leads to the release of degradation products I_2_, Br_2_, MAI, and MABr (Abdelmageed et al., 2016; Pistor et al., 2016; Wang et al., 2016; Ceratti et al., 2018). It was shown recently, that the accumulation of these compounds leads to the formation of RPM in perovskite films (Shlenskaya et al., 2018a; Udalova et al., 2020). Particularly, the fast formation of RPM under laser beam was confirmed for the six most relevant perovskite compositions: MAPbI_3_, MA_0.15_FA_0.85_PbI_3_, FAPbI_3_, FA_0.85_Cs_0.15_PbI_3_, MA_0.25_FA_0.75_PbI_2.25_Br_0.75_, and Cs_0.05_MA_0.15_FA_0.8_PbI_2.5_Br_0.5_ (Udalova et al., 2020).

Slow formation of RPM was observed under intense UV irradiation of a perovskite solar cell causing the degradation of the gold electrode through formation of [AuI_2_]^−^ and [AuI_4_]^−^ complexes and subsequent precipitation of the (MA)_2_Au_2_I_6_ phase on the Au/perovskite interface (Shlenskaya et al., 2018a). Ironically, methylammonium polybromides were also found to be responsible for perovskite self-healing under illumination (Ceratti et al., 2018) indicating a critical role of polyhalides in the photochemistry of hybrid perovskites.

## Conclusion and Outlook

To sum up, RPM are a new class of highly reactive polyhalide compounds with a general formula RHal_x_ (R = MA, FA, Cs; Hal = I, Br) attracting great interest in the view of their unusual physicochemical properties and potential applications. Demonstrating low melting temperatures and unprecedented phase diversity, these compounds can serve as facile precursors for perovskite solar cells fabrication and be responsible for their degradation and self-healing.

We believe that the RPM can promote the development of solvent-free methods of obtaining hybrid perovskites which is free from potential sources of contamination as the reaction between RPM and Pb proceeds without formation of any by-products. Hybrid perovskites obtained using RPM demonstrate excellent optoelectronic properties and have a large range of potential applications including solar cells, photodetectors, light-emitting diodes and photonic devices.

Today, further detailed physicochemical investigation of the RPM is needed to reveal the influence of various polyhalides on the functional properties of the perovskite and other accompanying materials in the devices. The objects of particular interest are RPM with mixed compositions and RPM with other cations including those which are able to form 2D perovskites and compounds with perovskite-like structure.

## Author Contributions

All authors listed have made a substantial, direct and intellectual contribution to the work, and approved it for publication.

## Conflict of Interest

The authors declare that the research was conducted in the absence of any commercial or financial relationships that could be construed as a potential conflict of interest.
